# Prevalence and associated factors of syphilis among female sex workers in East Africa: a systematic review and meta-analysis

**DOI:** 10.3389/fpubh.2025.1543119

**Published:** 2025-06-25

**Authors:** Solomon Gedfie, Woldeteklehymanot Kassahun, Abdu Jemal, Muluken Gashaw, Alembante Bazezew, Marye Nigatie, Getinet Kumie, Tadesse Misganaw, Zewdu Tefera, Bewuketu Belete Alemu, Bahriew Mezgebu, Brhanu Kassanew, Ephrem Tamrat, Wagaw Abebe, Agenagnew Ashagre, Assefa Sisay, Yalewayker Gashaw, Melese Abate Reta

**Affiliations:** ^1^Department of Medical Laboratory Sciences, College of Health Sciences, Woldia University, Woldia, Ethiopia; ^2^Department of Medical Microbiology, Faculty of Health Sciences, University of Pretoria, Pretoria, South Africa

**Keywords:** syphilis, *Treponema pallidum*, female sex workers, predictors, East Africa, meta analysis

## Abstract

**Background:**

Syphilis is the most common sexually transmitted infection caused by *Treponema pallidum*, a pathogen that is exclusive to humans. Syphilis is a highly treatable infection, but if left untreated, it can result in serious health complications, including adverse reproductive outcomes, diminished quality of life, and an increased risk of Human Immunodeficiency Virus (HIV) transmission. Female sex workers (FSWs) are considered a high-risk group for the transmission of syphilis. Therefore, this review aimed to estimate the pooled prevalence of syphilis and identify the associated factors among female sex workers in the East African region.

**Methods:**

This systematic review and meta-analysis was performed in accordance with the Preferred Reporting Items for Systematic Reviews and Meta-Analyses (PRISMA) guidelines. Databases such as PubMed/MEDLINE, Scopus, ScienceDirect, and Google Scholar search engines were explored to access eligible articles. STATA 11 statistical software was used to carry out the meta-analysis. A random-effects model was employed to estimate the pooled prevalence of syphilis and its predictors among female sex workers in the East African region. Higgen’s I^2^ test statistics was done to assess the heterogeneity of the included articles. Publication bias was evaluated visually using funnel plots and statistically through Egger’s weighted regression test.

**Results:**

A total of 16,456 articles were retrieved, among which 24 studies involving 25,979 female sex workers were included in the final meta-analysis. The pooled estimates of syphilis among female sex workers were 14.7% (95%CI: 11.06–18.35) and I^2^ of 99.1%, *p* = 0.000. Sub-group analyses were conducted based on country and publication year to address heterogeneity. The results revealed that the highest prevalence was 18.48% (95% CI: 11.064–25.899) in Ethiopia and 2.79% (95% CI: 1.49–4.09) in Kenya. Regarding publication year, the prevalence was 16.3% (95% CI: 12.01–20.61) in studies conducted before 2014 and 12.5% (95% CI: 5.85–19.16) in studies conducted after 2014. Among the factors old age was a significant predictor of syphilis among female sex workers.

**Conclusion:**

This review revealed a relatively higher prevalence of syphilis compared to the global estimate. To effectively curb syphilis among female sex workers in East Africa, intervention strategies should address the high prevalence and key associated factors through comprehensive approaches.

**Systematic Review Registration:**

https://www.crd.york.ac.uk/PROSPERO/view/CRD42024587246, CRD42024587246.

## Introduction

Syphilis is a common, preventable, and treatable sexually transmitted infection (STI) caused by the spirochete *Treponema pallidum*, which exclusively infects humans (1–4). Untreated syphilis can lead to severe complications, including adverse reproductive outcomes, decreased quality of life, and increased risk of HIV transmission (4, 5). Syphilis is transmitted through unprotected sex and vertically from mother to fetus during pregnancy or childbirth, resulting in congenital syphilis, a leading cause of fetal and neonatal morbidity and mortality worldwide (6, 7).

The prevalence of STIs and related complications remains a significant global health concern. Around 1 million people are infected with a curable STI each day (8, 9). The asymptomatic nature of most STIs, especially in women, often leaves individuals unaware of their infection, making control efforts more challenging. Individuals with STIs are at high risk for HIV acquisition (8, 10) and bad reproductive health outcomes including pelvic inflammatory disease (PID), ectopic pregnancy, infertility, and perinatal deaths (8, 11).

According to the Global Burden of Disease Report, STIs are the second leading cause of morbidity and mortality among women of reproductive age (12). It is a health concern in both developing and developed countries, with an estimated 340 million cases reported annually (13). The World Health Organization (WHO) reported that in over 10 countries, the prevalence of syphilis among FSWs was higher than 5% (14). In 2016, an estimated 376 million new cases of four curable STIs: chlamydia, gonorrhea, syphilis, and trichomoniasis were reported worldwide, averaging over 1 million new infections per day, with the prevalence rates differed across WHO regions (9).

The WHO reports approximately 12 million new cases of syphilis worldwide each year, with more than half of these cases occurring in Sub-Saharan Africa (15). Several studies have shown that syphilis prevalence is higher in Sub-Saharan Africa, particularly among groups engaged in high-risk sexual behaviors, such as commercial sex workers, drug users, and men who have sex with men (MSM) (16, 17). According to the WHO 2022 reports, the global average prevalence of active syphilis among FSWs is 10.8%, with rates varying significantly across regions, ranging from 5.8% to as high as 30.3% (18).

Female sex workers (FSWs) are considered a high-risk group for acquiring STIs like syphilis due to their nature of occupation. Factors such as social vulnerability, having multiple sexual partners, inconsistent condom use, and co-infections with other STIs significantly increase their risk of contracting the infection (1, 2). Prostitutes have been recognized by both the public and medical professionals as a high-risk group for the infection and transmission of STIs. While FSWs are frequently the focus of behavior change campaigns aimed at reducing STI transmission, this approach may oversimplify the complex interactions between the host, agent, and environment that contribute to STI spread (19).

Studies on the prevalence of syphilis infection among FSWs show considerable variation, with rates ranging from 1.5 to 42.1% (20). An earlier study also found that the risk of contracting syphilis was influenced not only by the socio-demographic profile and sexual behavior of FSWs but also by the type of sex work they engage in (21). For instance, among sub-groups of FSWs where workplace measures are in place to encourage condom use, the infection rate may be lower. In these cases, syphilis transmission is primarily linked to personal sexual partners, where consistent condom use is less frequent (22). It is also evident that prostitutes are not a homogeneous group; the personal circumstances and behaviors of some individuals may elevate their risk for both STIs and other health issues (23). Factors such as legal residency status in a country, duration of immigration, and citizenship rights, including access to healthcare, can impact the ability of FSWs to negotiate consistent condom use with their clients (24).

Individuals working in the sex industry, particularly those trafficked across borders illegally, face a significantly higher risk of STIs. Commercial heterosexual sex networks (CHSNs) are regarded as a major driver of STI transmission (25). The transmission rate of infections within a population can be affected by social, biological, cultural, and behavioral factors (2). STIs have been identified as proxy biomarkers for sexual risk behavior (26). They also serve as a valuable tool for analyzing the structure of sexual networks and predicting the potential for HIV epidemics (27). Programmatically, STI surveillance among FSWs remains insufficient, with infection rates frequently underreported. Furthermore, the sexual transmission of STIs within CHSNs is not well understood due to the lack of comprehensive and reliable self-reported sexual behavior data (28).

Although there are variable individual study findings, there is limited pooled epidemiological data on syphilis among FSWs in the East African region. In light of this, this systematic review and meta-analysis aimed to assess the pooled prevalence and associated factors of syphilis among FSWs in the East African region.

## Methods

### Protocol registration

This systematic review and meta-analysis was conducted in accordance with the PRISMA guidelines (29). This review’s protocol is registered in PROSPERO (registration number CRD42024587246).

### Searching strategy

Data were collected by searching published articles in publicly accessible electronic databases, including Scopus, PubMed/MEDLINE, ScienceDirect, and the Google Scholar search engine, and other institutional repositories and registries. There were no time restrictions for the article search; however, only articles published in English up to September 30, 2024, were included. The search terms were used separately and in combination using the Boolean operators of “OR” or “AND.” The search terms were “Syphillis,” “*Treponema pallidum*,” “Sexually transmitted diseases,” “Sexually transmitted infection,” “STI,” “STD,” “female sex workers,” “commercial sex workers,” “East-Africa.” The search strings used in the PubMed/MEDLINE was: ((((((((((((prevalence) OR (magnitude)) OR (epidemiology)) AND (syphilis)) OR (treponema pallidum)) OR (sexually transmitted infection)) OR (STI)) OR (STD)) AND (Female sex workers)) OR (commercial sex workers)) OR (prostitute)) AND (East-Africa)) OR Comoros[tw] OR Djibouti[tw] OR Eritrea[tw] OR Ethiopia[tw] OR Madagascar[tw] OR Malawi[tw] OR Mali[tw] OR Mauritania[tw] OR Mauritius[tw] OR Mayote[tw] OR Mozambique[tw] OR Mocambique[tw] OR Reunion[tw] OR Rwanda[tw] OR Seychelles[tw] OR Somalia[tw] OR Tanzania[tw] OR Uganda[tw] OR “East Africa”[tw] OR “East African”[tw] OR “Eastern Africa”[tw] OR “Eastern African”[tw]. Additional publications were searched by manual search and by looking into references in pertinent papers. The search strategy and number of articles retrieved from the searched databases and additional searches are depicted in the additional file (Supplementary Table S1).

### Inclusion and exclusion criteria

This review includes articles published in peer-reviewed journals and deposited in institutional electronic library repositories or registries, which report the prevalence of syphilis among FSWs in the East African region. Eligible studies include case–control, cross-sectional, and cohort studies that report the outcome of interest. Only articles published in English before September 30, 2024, were considered for inclusion. Case reports, case series, conference papers, and non-English papers were excluded.

### Outcomes on interest

The outcome of interest for this systematic review and meta-analysis was the pooled prevalence and associated factors of syphilis among FSWs in the East African region.

### Study selection and quality assessment

To organize the search results and remove duplicate studies, the retrieved articles were imported into EndNote X21 (Thomson Reuters, New York, United States). The article searches from electronic databases, and additional searches from registries and Google Scholar were conducted by three reviewers (SG, WTK, and ZT). The articles were first screened by titles independently by two reviewers (SG, AJ), and abstract screening was conducted by three reviewers (MN, AA, and BK) independently. Full-text screening was carried out by four reviewers (SG, AB, BM, and MG) independently. Any disagreements between reviewers were resolved through discussion and the involvement of additional reviewers (WA, BBA, and AS). Additionally, the methodological quality of the included studies was assessed using the Hoy risk of bias tool, based on the Jonna Bridge Institute (JBI) critical appraisal tools by reviewers (MAR, TM, GK, and YG) (30) (Supplementary Table S2). The quality appraisal guideline consists of 10 evaluation domains or categories to assess both the internal and external validity of the studies. These items are: (a) representativeness of the population, (b) sampling frame, (c) methods of study unit selection, (d) bias due to non-response, (e) data source (primary data), (f) acceptability of case definition, (g) reliability and validity of the study tool, (h) mode of data collection, (i) appropriateness of the numerator and denominator, and (j) summary. Each category was rated as either low or high risk of bias, with unclear categorized as high risk. The overall risk of bias for each study was determined based on the number of high-risk categories, with studies classified as low (0–3), moderate (4–6), or high (7–9) risk of bias (Supplementary Table S2).

### Data extraction

Data extraction was performed independently by two reviewers (SG and MN) from studies meeting the eligibility criteria. The extracted information was compiled into an MS Excel spreadsheet. Any disagreements were resolved through consensus and discussions involving additional reviewers (MAR, MN, and ET). The following items were extracted for analysis: the first author’s name, year of publication, total number of participants, prevalence of the outcome variable, participants’ age, study design, odds ratios of predictors with 95% confidence intervals, and the country where the study was conducted (Table 1).

**Table 1:** Characteristics of included studies in the meta-analysis of syphilis among FSWs in East Africa.

Author	Year of publication	country	Study design	Sample size	No of cases	Mean age	Prevalence	Factor_1	Odds ratio_1	Factor_2	Odds ratio_2
Ahmed et al. (58)	1991	Somalia	Prospective cohort	155	107	26(15–55)	69%				
Desta et al. (34)	1990	Ethiopia	Cross-sectional	203	76	23.4 years	37.4%	Duration in sex work			
Geyid et al. (35)	1990	Ethiopia	Cross-sectional	193	72		37.31%				
Behets et al. (46)	2005	Madagascar	Cross-sectional	1,285	76	(16–57)	5.91%				
Metaferia et al. (36)	2021	Ethiopia	Cross-sectional	360	45	(18–27)	12.5%	Being Single	0.044 (0.003–0.622)	Divorced	0.037 (0.003–0.501)
Tura et al. (37)	2023	Ethiopia	Cross-sectional	6,085	378	20–24	6.2%	No formal education	3.38 (2.34–5.11)	divorced	1.37 (1.03–1.82)
Hakim et al. (53)	2020	South Sudan	Cross-sectional	838	62	>15	7.3%				
Manguro et al. (40)	2013	Kenya	Cross-sectional	479	16	(20–30)	3.34%				
Fonck et al. (41)	2001	Kenya	Cross-sectional	528	31	30 (7.8SD)	5.87%				
Wariso et al. (38)	2023	Ethiopia	Cross-sectional	6,085				selling sex above 11 years	1.21 (1.03–1.43)	aged 25–34 years	2.99 (2.54–3.52)
Harijaona et al. (47)	2009	Madagascar	Cross-sectional	100	11	(15–29)	11%	Marital status Single	0.2 (0.1–0.5)	No. Primigravida	0.3 (0.1–0.9)
Hawken et al. (42)	2002	Kenya	Cross-sectional	503	11	(15–65)	2%				
Nzivo (43)	2019	Kenya	Cross-sectional	268	6		2.24%				
Okiria et al. (54)	2023	South Sudan	Cross-sectional	409	38	(23–35)	9.2%	HIV infection	6.99 (2.23–21.89)		
Musyoki et al. (44)	2014	Kenya	Cross-sectional	596	6	(25–38)	0.9%				
Vandepitte et al. (49)	2011	Uganda	Cross-sectional	1,027	216	Undefined	21%				
Tukamwesiga (50)	2017	Uganda	Cross-sectional	86	10	20–30	10.5%.				
G Riedner Riedner et al. (51)	2015	Tanzania	Cross-sectional	600	56	Undefined	9%				
Mutagoma et al. (55)	2017	Rwanda	Cross-sectional	1978	1,011	20–63	51.1%				
Alemu et al. (39)	2022	Ethiopia	Cross-sectional	381	17	20–24	4.2%				
Xueref et al. (48)	2003	Madagascar	Cross-sectional	316	59		18.4%				
Vu and Misra (52)	2017	Tanzania	Cross-sectional	1914	154		8%				
Chersich et al. (45)	2007	Kenya	Cross-sectional	692	22	30.4 years	3.2%	Unprotected sex	1.59 (1.00–2.53)	Sexual violence	1.85 (1.27–2.71)
Kotlewski et al. (56)	2014	Zambia	Cross-sectional	733	93		12.62%				
Choongo (57)	2019	Zambia	Cohort	165	30	18–48	17.58%	History of Syphilis	14.52 (5.63–37.45)		

### Statistical analysis

The recorded data were reviewed for completeness in a Microsoft Excel sheet and subsequently imported into STATA version 11 statistical software for final analysis. Initially, a fixed-effects model was used for the analysis. However, substantial heterogeneity was detected during the heterogeneity assessment. Consequently, a random-effects model was applied to estimate the effect size with a 95% confidence interval (95% CI) (31). Forest plots were used to display the overall effect size and the weight contributed by each study, along with a 95%CI. Graphic representations of the pooled estimates were also included. The degree of heterogeneity among the studies was assessed using Higgins’ I^2^ statistic (32). I^2^ values of 75, 50, and 25% were interpreted as indicators of high, medium, and low heterogeneity, respectively. Subgroup analysis was performed based on the country where the study was conducted and the year of publication to identify potential sources of heterogeneity. Sensitivity analysis was conducted to determine whether any individual study exerted a dominant influence over the results. This was achieved by systematically omitting each study one at a time. Publication bias was assessed using Egger’s weighted regression test and Funnel plots. A *p*-value of less than 0.05 in Egger’s test was considered indicative of significant publication bias (33).

## Results

### Searching results

**Figure 1:**
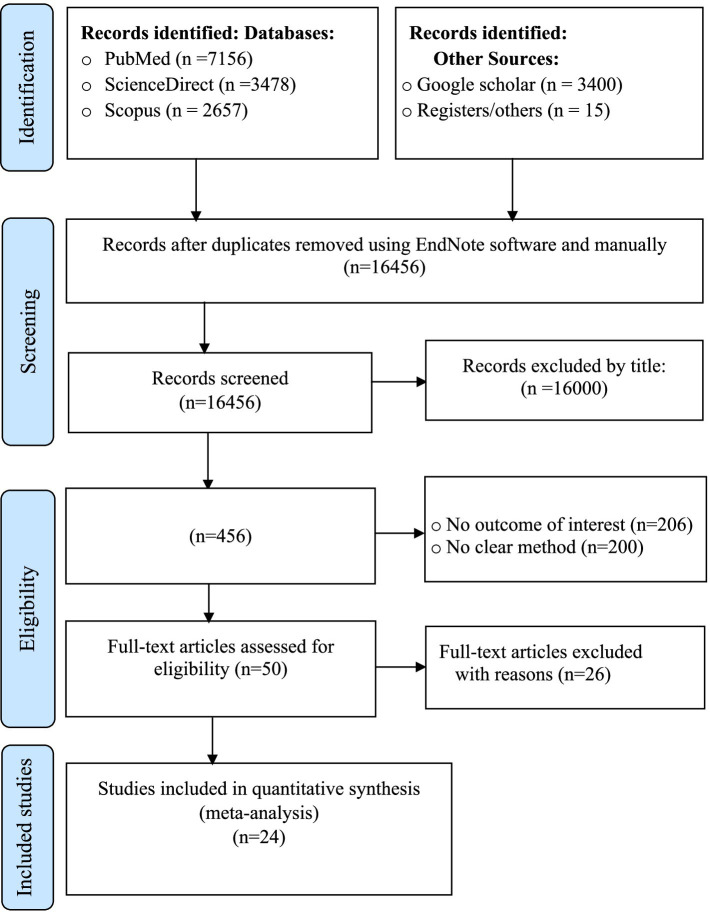
PRISMA flow diagram showing the results of the search and reasons for exclusion on systematic review and meta-analysis of syphilis among FSWs in East Africa (29).

This systematic review and meta-analysis focused on published studies examining syphilis among female sex workers (FSWs) in East Africa. The databases utilized in the search strategy included PubMed/MEDLINE, ScienceDirect, Scopus, and Google Scholar search engine. The initial search identified 16,706 studies. Of these, 200 records were excluded due to duplication, and 16,456 were excluded after screening titles and abstracts. A total of 50 full-text articles were assessed for eligibility, of which 26 were excluded for reporting only other STIs instead of syphilis. Finally, 24 potential studies were included in the final quantitative meta-analysis (Figure 1).

### Characteristics of included studies

A total of 24 studies were included in this systematic review and meta-analysis, of which six were conducted in Ethiopia (34–39), six in Kenya (40–45), three in Madagascar (46–48), two in Uganda (49, 50), two in Tanzania (51, 52), two in South Sudan (53, 54), one in Rwanda (55), two in Zambia (56, 57) and one from Somalia (58). The sample size ranges from 86 in Uganda (50) to 6,085 in Ethiopia (37). The highest prevalence of syphilis was reported from Somalia (58) (69%), whereas the lowest prevalence was reported from Kenya (44) (0.9%; Table 1).

### Publication bias

**Figure 2:**
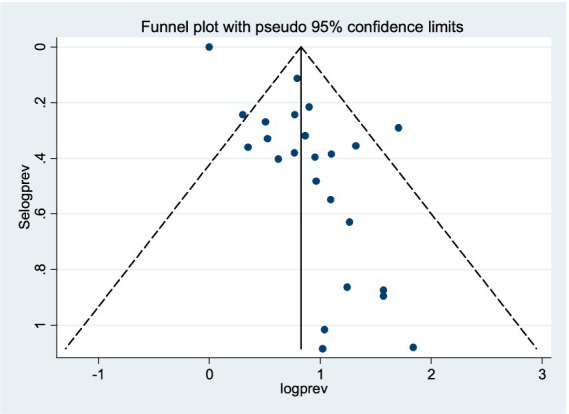
Funnel plot on the prevalence of syphilis among FSWs in East Africa.

The included studies were assessed for publication bias through a visual examination of the funnel plot and Egger’s test statistics. From the funnel plot analysis, we observed that the funnel plot appeared asymmetric, indicating evidence of publication bias (Figure 2). Furthermore, Egger’s weighted regression test confirmed the presence of potential publication bias among the included studies (*p* = 0.010; Table 2). Based on the evidence from both the visual inspection of the funnel plot and Egger’s test statistics, a trim-and-fill analysis was conducted to assess the bias caused by the small study effect. Following the trimming and filling, the result indicates 5.387% (95%CI: 1.343–9.430).

**Table 2:** Egger’s test for syphilis prevalence among FSWs in East Africa.

Std_Eff	Coef.	Std. Err.	*t*	P > t	95% Conf. interval	
Slope	0.4356754	2.632096	0.17	0.870	−5.022958	5.894308
Bias	9.212829	3.272175	2.82	0.010	2.426753	15.99891

CI, Confidence interval; Std Eff, Standard effect; Coef, coefficient; Std. Err, Standard error.

### Trim and fill analysis

To address the presence of publication bias, a trim-and-fill analysis was performed to mitigate the impact of the small-study effect. Ten additional studies were added to the model, resulting in an estimated pooled prevalence of syphilis among FSWs of 5.387% (95% CI: 1.343–9.430) using the random-effects model.

### Sensitivity analysis

A sensitivity analysis was conducted using a random-effects model to assess the impact of each study on the pooled estimate of syphilis. The analysis involved systematically omitting one study at a time. The results indicated that the omission of any single study did not significantly affect the overall pooled estimate of syphilis (Table 3).

**Table 3:** Sensitivity analysis of included studies on the pooled estimate of syphilis among FSWs in East Africa.

Study omitted	Point | estimate	95% confidence interval (CI)	
		Lower CI	Upper CI
Ahmed et al. (58)	12.576372	9.057394	16.09535
Desta et al. (34)	13.789755	10.12174	17.457769
Geyid et al. (35)	13.800068	10.129585	17.470551
Behets et al. (46)	15.134671	11.255639	19.013704
Metaferia et al. (36)	14.798603	11.057208	18.539997
Tura et al. (37)	15.215841	10.901551	19.53013
Hakim et al. (53)	15.052913	11.243226	18.8626
Manguro et al. (40)	15.23305211	417,819	19.048286
Fonck et al. (41)	15.111306	11.319636	18.902977
Harijaona et al. (47)	14.849442	11.121594	18.57729
Hawken et al. (42)	15.305374	11.450074	19.160675
Nzivo (43)	15.275846	11.482792	19.068899
Okiria et al. (54)	14.950356	11.191838	18.708874
Musyoki et al. (44)	15.37806	11.433524	19.322596
Vandepitte et al. (49)	14.402218	10.727677	18.076757
Tukamwesiga (50)	14.867318	11.140656	18.593979
G Riedner Riedner et al. (51)	14.965387	11.191434	18.73934
Mutagoma et al. (55)	12.309308	9.9399967	14.67862
Alemu et al. (39)	15.185042	11.397274	18.972811
Xueref et al. (48)	14.538399	10.818944	18.257853
Vu and Misra (52)	15.044206	11.1507	18.937712
Chersich et al. (45)	15.251049	11.39506	19.107038
Kotlewski et al. (56)	14.798405	11.043707	18.553104
Choongo (57)	14.580793	10.859785	18.301802
Combined	14.69879	11.049455	18.348124

### Pooled prevalence of syphilis among FSWs in East Africa

**Figure 3:**
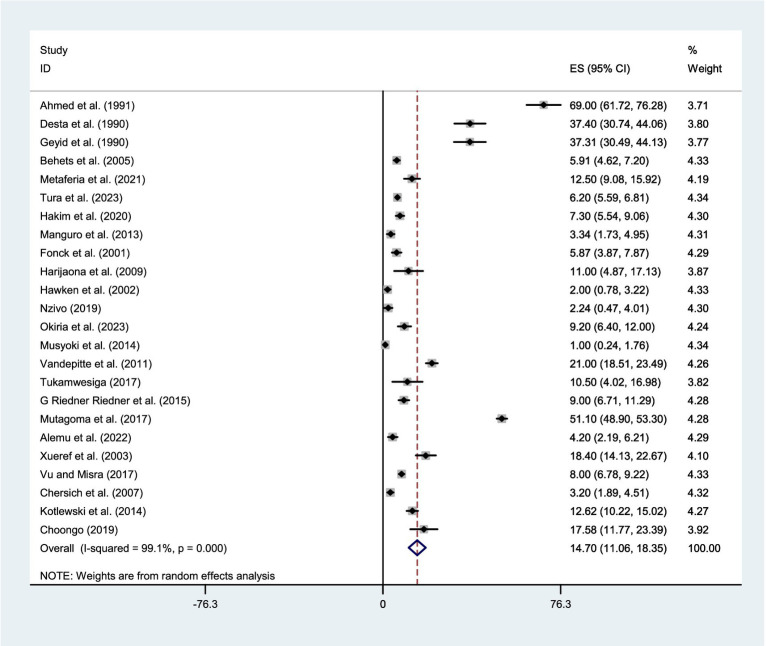
Forest plot showing the pooled prevalence of syphilis among FSWs in East Africa, using the random effect model.

The pooled estimate of syphilis among FSWs in East Africa by the fixed-effect model was 6.496% (95% CI: 6.174–6.818) and I^2^ of 99.1%, *p* = 0.000. Therefore, a *p*-value less than 0.05 and I^2^ of 99.1% implies the existence of substantial heterogeneity. Finally, by considering this heterogeneity, a random-effect model was applied. Based on the Der Simonian-Laird random-effects model, the pooled estimate of syphilis was 14.702% (95% CI: 11.057–18.346) and I^2^ of 99.1%, *p* = 0.000 (Figure 3).

**Figure 4:**
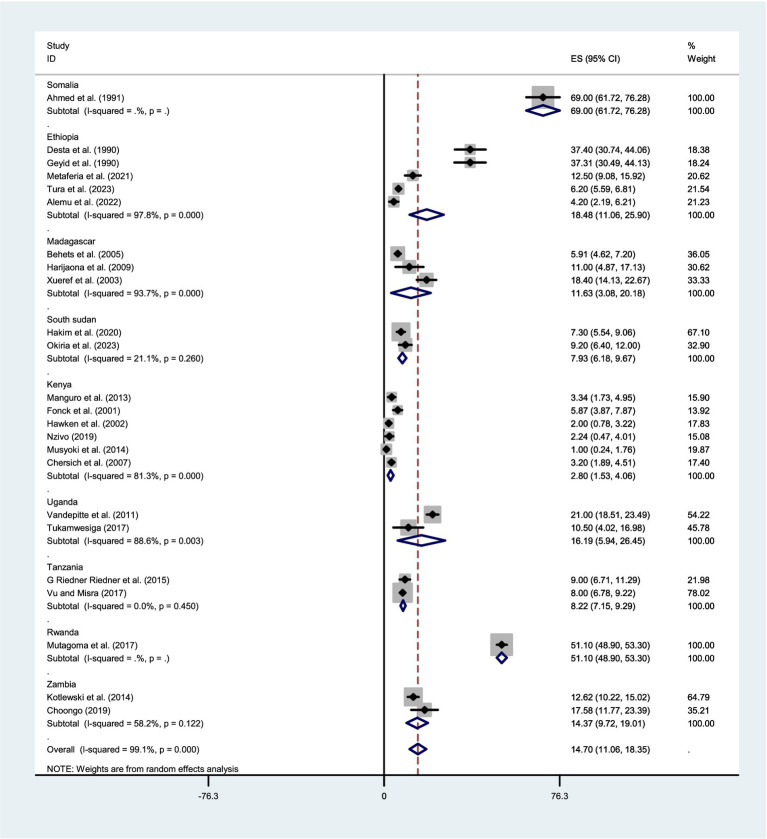
Forest plot showing sub-group analysis of the pooled prevalence of syphilis among FSWs in the East-Africa by Country.

**Figure 5:**
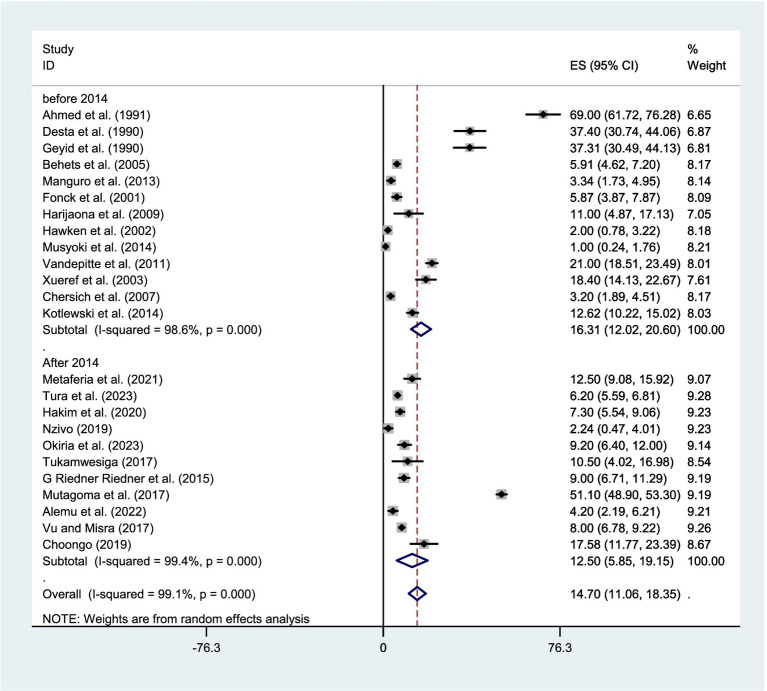
Forest plot showing sub-group analysis of the pooled prevalence of syphilis among FSWs in East Africa by year of publication.

Given the significant evidence of heterogeneity across studies (I^2^ = 99.1%, *p* < 0.001), a subgroup analysis was conducted based on country and year of publication. The studies were stratified into two groups according to publication year: those published before 2014 and those published in 2014 or later. Therefore, result revealed that the pooled estimate of syphilis among FSWs was 18.48% (95% CI:11.064–25.899) in Ethiopia, 11.631% (95% CI: 3.08–20.18) in Madagascar, 7.93% (95% CI: 6.18–9.68) in South Sudan, 2.79% (95%CI:1.485–4.087) in Kenya, 16.193% (95%CI: 5.94–26.446) in Uganda, 8.22% (95%CI: 7.146–9.293) in Tanzania, and 14.37% (95% CI: 9.72–19.01) in Zambia (Figure 4). Whereas, based on the year of publication, the pooled estimate of syphilis was 16.31% (95% CI: 12.01–20.61) among studies conducted before 2014 and 12.50% (95% CI: 5.852–19.155) among studies conducted after 2014 (Figure 5). Two subgroups were analyzed to assess the impact of geographical location and the local level of awareness about syphilis transmission, as well as access to information about the disease, which may contribute to heterogeneity across the countries. Additionally, differences in the year of publication were considered, as they may reflect trends in lifestyle changes among the study participants.

### Factors associated with the prevalence of syphilis among FSWs

**Figure 6:**
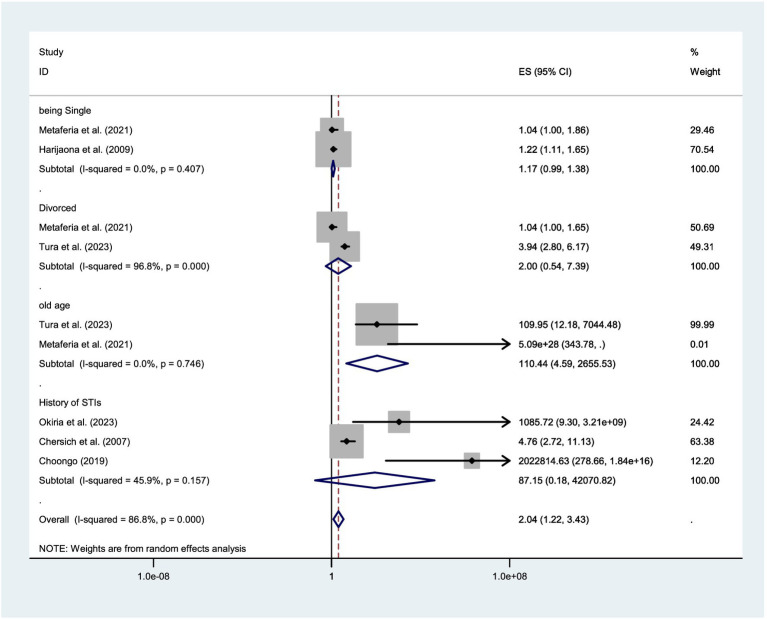
Forest plot showing factors associated with the prevalence of syphilis among FSWs in the East African regions.

For the pooled estimates of factors associated with syphilis among FSWs in East Africa, this meta-analysis included two studies examining the association with being single (36, 47), two studies assessing the impact of older age (36, 37), and three studies evaluating the effect of a history of STIs (45, 54, 57). Among the various predictors assessed in this meta-analysis, only older age emerged as a statistically significant predictor of syphilis FSWs (Figure 6).

## Discussion

Female sex workers (FSWs) are at a high risk for both acquiring and transmitting sexually transmitted infections, particularly syphilis. However, evidence on the epidemiology and risk factors of syphilis among FSWs in East Africa is limited. Therefore, this systematic review and meta-analysis aimed to determine the pooled prevalence of syphilis among FSWs in the East African region.

The pooled prevalence of syphilis among FSWs in East Africa was found to be 14.70%. The estimate is comparable to findings from other regions. For example, a systematic review and meta-analysis conducted in the Middle East and North Africa reported a pooled prevalence of 12.58% (59), while studies from Brazil and Southern China documented prevalence rates of 14.1% (60) and 15.7% (61), respectively. Additionally, this result aligns with an individual study conducted in Hefei, China, which reported a prevalence of 17.3% (62).

In contrast, the findings of this systematic review and meta-analysis indicate a higher prevalence of syphilis among FSWs compared to several studies conducted in other countries. For instance, studies reported lower prevalence rates in Iran (0.33%) (63), China (2.91–9.67%) (64–67), Brazil (2.4–8.5%) (68, 69), and Togo (2.20%) (70). The observed differences in prevalence rates may be attributed to variations in sample sizes, methodological approaches, and the socio-demographic and behavioral profiles of the populations involved in each study. Furthermore, the variation could be partly attributed to differing levels of public awareness regarding syphilis and disparities in the coverage, accessibility, and quality of medical screening and intervention efforts across the study settings. In some regions, limited public health education and insufficient screening programs may lead to underreporting, whereas areas with robust awareness initiatives and widespread testing could detect more cases and reporting. This disparity highlights the need for targeted health campaigns and improved access to diagnostic services to ensure accurate case detection and reporting. Moreover, factors such as war/conflict, recurrent drought, and inconsistent screening services in East Africa may have impeded early detection and treatment efforts, thereby contributing to the higher prevalence of syphilis among FSWs in the region.

Conversely, the finding of this review was lower than those reported in several individual studies. For example, a study conducted in Brazil reported a prevalence of 36.94% (71), while an international review documented prevalence rates ranging from 1.5–60.5%, with particularly higher rates observed in sub-Saharan Africa, North Africa and the Middle East, Latin America, and South Asia, where prevalence ranged from 25 to 60% (72). Similarly, higher prevalence rates were reported in China (43.5%) (73), and Rwanda (51.1%) (55). The observed disparities may be attributed to variations in sample size, geographic context, disease awareness, and access to screening and treatment services. In East Africa, the discreet nature of sex work and limited engagement with public health services may contribute to underreporting and a lower detected prevalence compared to other regions.

Due to the evidence of heterogeneity across studies, a subgroup analysis was conducted based on the country and year of publication. The results showed that the pooled prevalence of syphilis among FSWs varied by country: Ethiopia (18.48%), Madagascar (11.6%), South Sudan (7.9%), Kenya (2.8%), Uganda (16.2%), Tanzania (8.2%), and Zambia (14.4%). The observed variations could be explained by differences in study sample sizes, geographic contexts, levels of syphilis awareness among FSWs, and the degree of government prioritization of FSWs’ sexual health. Regions with larger sample sizes, higher awareness, and stronger public health interventions may report more accurate prevalence rates, while areas with limited testing and healthcare access could show underestimated figures. The discrepancy could also stem from variations in the number of studies analyzed in each region and differences in the intensity of syphilis screening campaigns. Areas with more comprehensive research and active screening programs may report higher detection rates, while regions with fewer studies and limited testing efforts might show lower prevalence.

Subgroup analysis by publication year showed a higher syphilis pooled prevalence of 16.31% in studies published before 2014, compared to 12.50% in those published after 2014, suggesting a possible decline in prevalence over time. This trend could reflect improvements in screening, treatment accessibility, or public health interventions in recent years. While global trends indicate rising syphilis rates among key populations in developed and middle-income countries, our review observed a declining trend. This discrepancy may be attributed to variations in the number of studies/evidence, sample sizes, and reported cases between different regions and periods. The limited number of included studies in certain subgroups could influence the overall pooled estimates, potentially masking underlying epidemiological patterns. Additionally, the observed decline could reflect expanded antiretroviral therapy access, which often includes integrated STI screening, counseling, and prevention education. Alternatively, improved health awareness and strengthened regular screening campaigns may have contributed to reduced syphilis prevalence.

Among the factors identified only old age was a significant predictor of syphilis among FSWs. This evidence is supported by individual reports from Ethiopia (36, 37) reporting that older age is a risk factor for acquiring syphilis among FSWs. This suggests that the risk of syphilis infection increases with age in this population, potentially due to prolonged exposure to high-risk sexual behaviors, cumulative occupational hazards, or limited access to consistent preventive healthcare services over time.

The high syphilis prevalence among FSWs stems from interconnected behavioral, biological, and socioeconomic factors. Key behavioral risks include unprotected sex, condom failure, and multiple partners. Biological factors encompass prior STIs, previous syphilis infection, and sexual violence. Socioeconomic vulnerabilities like low education and multiparty exacerbate risks by creating structural barriers. These elements collectively facilitate transmission while undermining prevention through reduced self-efficacy, compromised protective behaviors, and perpetuated socioeconomic marginalization that limits healthcare access.

## Limitation and strength

A key limitation of this systematic review and meta-analysis is the uneven representation of data across East African countries, with some nations contributing more studies than others, potentially skewing subgroup analyses by country. Additionally, the exclusion of non-English-language studies due to a lack of translation resources may have introduced language bias. However, a major strength of this study is its rigorous methodology, including a comprehensive search across multiple databases using varied search strategies to minimize selection bias. This thorough approach enhances the reliability and generalizability of the findings despite the noted limitations.

## Conclusion

This systematic review and meta-analysis reveals an intermediate pooled prevalence of syphilis among FSWs in East Africa. As FSWs represent a high-risk population and key drivers of STI transmission in the region, these findings underscore the need for targeted interventions. To effectively address this public health challenge, comprehensive strategies should be implemented, including routine screening programs, risk reduction counseling, and sustained prevention efforts tailored to local contexts across East African countries. Such measures are critical for reducing syphilis transmission and its associated health burdens in this vulnerable population.

## Data Availability

The original contributions presented in the study are included in the article/Supplementary material, further inquiries can be directed to the corresponding author.
