# Supplementary material for: Prevalence and associated factors of syphilis among female sex workers in East Africa: a systematic review and meta-analysis
Source: Front Public Health. 2025 Jun 25;13:1543119. doi: 10.3389/fpubh.2025.1543119 (PMC12238060; doi:10.3389/fpubh.2025.1543119)
Supplement: Supplementary file 1 [file Data_Sheet_1.docx]

**Prevalence and associated factors of syphilis among female sex workers in East Africa: A systematic review and meta-analysis**

**Supplementary Files**

**Table S1:** Search databases for prevalence and associated factors of syphilis among female sex workers in East Africa

| **S.No-** | **Database** | **Search string** | **Total number of article** |
| --- | --- | --- | --- |
| 1 | PubMed | The search strings used in the PubMed/MEDLINE was:- ((((((((((((prevalence) OR (magnitude)) OR (epidemiology)) AND (syphilis)) OR (treponema pallidum)) OR (sexually transmitted infection)) OR (STI)) OR (STD)) AND (Female sex workers)) OR (commercial sex workers)) OR (prostitute)) AND (East-Africa)) OR Comoros[tw] OR Djibouti[tw] OR Eritrea[tw] OR Ethiopia[tw] OR Madagascar[tw] OR Malawi[tw] OR Mali[tw] OR Mauritania[tw] OR Mauritius[tw] OR Mayote[tw] OR Mozambique[tw] OR Mocambique[tw] OR Reunion[tw] OR Rwanda[tw] OR Seychelles[tw] OR Somalia[tw] OR Tanzania[tw] OR Uganda[tw] OR "East Africa"[tw] OR "East African"[tw] OR "Eastern Africa"[tw] OR "Eastern African"[tw]. | 7156 |

**Table S2**: The methodological quality of the included studies using JBI critical appraisal tools(cross-sectional studies)

| Author | Were the criteria for inclusion in the sample clearly  defined? | Were the study subjects and the setting described in  detail? | Was the exposure measured in a valid and reliable  way? | Were objective, standard criteria used for  measurement of the condition? | Were confounding factors identified? | Were strategies to deal with confounding factors  stated? | Were the outcomes measured in a valid and reliable  way? | Was appropriate statistical analysis used? | Summary of the  overall  result |
| --- | --- | --- | --- | --- | --- | --- | --- | --- | --- |
| Desta *et al.,* 1990 (1) | 1 | 1 | 1 | 1 | 1 | 1 | 1 | 1 | 8 |
| Ahmed *et al*., 1991  (2) | 1 | 1 | 1 | 1 | 0 | 0 | 1 | 1 | 6 |
| Geyid *et al.,* 1990 (3) | 1 | 1 | 1 | 1 | 1 | 0 | 1 | 1 | 7 |
| Behets *et al.,* 2005  (4) | 1 | 1 | 1 | 1 | 0 | 0 | 1 | 1 | 6 |
| Metaferia *et al.,* 2021(5) | 1 | 1 | 1 | 1 | 1 | 1 | 1 | 1 | 8 |
| Tura *et al.,* 2023 (6) | 1 | 1 | 1 | 1 | 1 | 1 | 1 | 1 | 8 |
| Hakim *et al.,* 2020(7) | 1 | 1 | 1 | 1 | 0 | 0 | 1 | 1 | 6 |
| Manguro *et al.,* 2013(8) | 1 | 1 | 1 | 1 | 0 | 0 | 1 | 1 | 6 |
| Fonck *et al., 2001*(9) | 1 | 1 | 1 | 1 | 0 | 0 | 1 | 1 | 6 |
| Wariso *et al.,* 2023(10) | 1 | 1 | 1 | 1 | 1 | 1 | 1 | 1 | 8 |
| Harijaona *et al.,* 2009 (11) | 1 | 1 | 1 | 1 | 1 | 1 | 1 | 1 | 8 |
| Hawken *et al.,* 2002 (12) | 1 | 1 | 1 | 1 | 0 | 0 | 1 | 1 | 6 |
| Nzivo, 2019 (13) | 1 | 1 | 1 | 1 | 0 | 0 | 1 | 1 | 60 |
| Okiria *et al.,* 2023 (14) | 1 | 1 | 1 | 1 | 1 | 1 | 1 | 1 | 8 |
| Musyoki *et al.,* 2015 (15) | 1 | 1 | 1 | 1 | 1 | 0 | 1 | 1 | 7 |
| Vandepitte *et al.,* 2011 (16) | 1 | 1 | 1 | 1 | 0 | 0 | 1 | 1 | 6 |
| Tukamwesiga, 2017 (17) | 1 | 1 | 1 | 1 | 0 | 0 | 1 | 1 | 6 |
| G Riedner Riedner *et al.,* 2003  (18) | 1 | 1 | 1 | 1 | 0 | 0 | 1 | 1 | 6 |
| Mutagoma *et al*., 2017(19) | 1 | 1 | 1 | 1 | 0 | 0 | 1 | 1 | 6 |
| Alemu *et al.,* 2022 (20) | 1 | 1 | 1 | 1 | 1 | 0 | 1 | 1 | 7 |
| Xueref *et al.,* 2003 (21) | 1 | 1 | 1 | 1 | 0 | 0 | 1 | 1 | 6 |
| Vu and Misra, 2018  (22) | 1 | 1 | 1 | 1 | 0 | 0 | 1 | 1 | 6 |
| Chersich *et al.,* 2007  (23) | 1 | 1 | 1 | 1 | 1 | 1 | 1 | 1 | 8 |
| Kotlewski *et al.,* 2014 (24) | 1 | 1 | 1 | 1 | 0 | 0 | 1 | 1 | 6 |
| Choongo, 2019 (25) | 1 | 1 | 1 | 1 | 0 | 0 | 1 | 1 | 6 |

**References**

1. Desta S, Feleke W, Yusuf M, Mehret M, Geyid A, Ghidinllie M, et al. Prevalence of STD and STD related risk factors in sex workers of Addis Ababa. Ethiopian Journal of Health Development. 1990;4(2).

2. Ahmed HJ, Omar K, Adan SY, Guled AM, Grillner L, Bygdeman S. Syphilis and human immunodeficiency virus seroconversion during a 6-month follow-up of female prostitutes in Mogadishu, Somalia. International journal of STD & AIDS. 1991;2(2):119-23.

3. Geyid A, Tesfaye HS, Abraha A, Lemeneh Y, Desta S, Feleke W. Isolates of STDs causative agents from sex workers Addis Ababa (a preliminary report). Ethiopian Journal of Health Development. 1990;4(2).

4. Behets FM, Van Damme K, Rasamindrakotroka A, Hobbs M, McClamroch K, Rasolofomanana JR, et al. Socio-demographic and behavioural factors associated with high incidence of sexually transmitted infections in female sex workers in Madagascar following presumptive therapy. Sexual health. 2005;2(2):77-84.

5. Metaferia Y, Ali A, Eshetu S, Gebretsadik D. Seroprevalence and associated factors of human immunodeficiency virus, treponema pallidum, hepatitis B virus, and hepatitis C virus among female sex workers in Dessie City, Northeast Ethiopia. BioMed research international. 2021;2021(1):6650333.

6. Tura JB, Ayalew J, Moreda AB, Lulseged S, Rameto MA, Debel LN, et al. Prevalence of syphilis and associated factors among female sex workers in Ethiopia: findings from a multilevel analysis of a national bio-behavioral survey. BMC public health. 2023;23(1):809.

7. Hakim AJ, Bolo A, Werner M, Achut V, Katoro J, Caesar G, et al. High HIV and syphilis prevalence among female sex workers in Juba, South Sudan. PloS one. 2020;15(9):e0239543.

8. Manguro GO, Gichuki C, Ampt FH, Agius PA, Lim MS, Jaoko WG, et al. HIV infections among female sex workers in Mombasa, Kenya: current prevalence and trends over 25 years. International journal of STD & AIDS. 2020;31(14):1389-97.

9. Fonck K, Kaul R, Keli F, Bwayo JJ, Ngugi EN, Moses S, et al. Sexually transmitted infections and vaginal douching in a population of female sex workers in Nairobi, Kenya. Sexually transmitted infections. 2001;77(4):271-5.

10. Wariso FB, Ayalew J, Barba A, Bedassa BB, Ebo GG, Tura JB, et al. Determinants of sexually transmitted infections among female sex workers in Ethiopia: a count regression model approach. Frontiers in public health. 2023;11:1190085.

11. Harijaona V, Ramambason J, Morisset R, Rasamindrakotroka A, Ravaoarinoro M. Prevalence of and risk factors for sexually-transmitted infections in hidden female sex workers. Médecine et maladies infectieuses. 2009;39(12):909-13.

12. Hawken MP, Melis R, Ngombo D, Mandaliya K, Price J, Dallabetta G, et al. Part time female sex workers in a suburban community in Kenya: a vulnerable hidden population. Sexually transmitted infections. 2002;78(4):271-3.

13. Nzivo MM. Human herpes virus types 8 and its coinfection with human immunodeficiency virus and treponema pallidum among female sex workers in Malindi, Kenya 2019.

14. Okiria AG, Achut V, McKeever E, Bolo A, Katoro J, Arkangelo GC, et al. High HIV and syphilis prevalence among female sex workers and sexually exploited adolescents in Nimule town at the border of South Sudan and Uganda. PloS one. 2023;18(1):e0266795.

15. Musyoki H, Kellogg TA, Geibel S, Muraguri N, Okal J, Tun W, et al. Prevalence of HIV, sexually transmitted infections, and risk behaviours among female sex workers in Nairobi, Kenya: results of a respondent driven sampling study. AIDS and behavior. 2015;19:46-58.

16. Vandepitte J, Bukenya J, Weiss HA, Nakubulwa S, Francis SC, Hughes P, et al. HIV and other sexually transmitted infections in a cohort of women involved in high-risk sexual behavior in Kampala, Uganda. Sexually transmitted diseases. 2011;38(4):316-23.

17. Tukamwesiga N. Prevalence of syphilis among commercial sex workers in Mbarara Municipality. 2017.

18. Riedner G, Rusizoka M, Hoffmann O, Nichombe F, Lyamuya E, Mmbando D, et al. Baseline survey of sexually transmitted infections in a cohort of female bar workers in Mbeya Region, Tanzania. Sexually transmitted infections. 2003;79(5):382-7.

19. Mutagoma M, Nyirazinyoye L, Sebuhoro D, Riedel DJ, Ntaganira J. Syphilis and HIV prevalence and associated factors to their co-infection, hepatitis B and hepatitis C viruses prevalence among female sex workers in Rwanda. BMC infectious diseases. 2017;17:1-9.

20. Alemu GH, Gidebo DD, Ali MM. SEROPREVALENCE OF SYPHILIS AMONG FEMALE COMMERCIAL SEX WORK-ERS IN HAWASSA, ETHIOPIA: A CROSSECTIONAL STUDY. Reason. 2022;11:2.9.

21. Xueref S, Holianjavony J, Daniel R, Kerouedan D, Fabry J, Vanhems P. The absence of HIV seropositivity contrasts with a high prevalence of markers of sexually transmitted infections among registered female sex workers in Toliary, Madagascar. Tropical Medicine & International Health. 2003;8(1):60-6.

22. Vu L, Misra K. High burden of HIV, syphilis and HSV-2 and factors associated with HIV infection among female sex Workers in Tanzania: implications for early treatment of HIV and pre-exposure prophylaxis (PrEP). AIDS and behavior. 2018;22:1113-21.

23. Chersich M, Luchters S, Malonza I, Mwarogo P, King'Ola N, Temmerman M. Heavy episodic drinking among Kenyan female sex workers is associated with unsafe sex, sexual violence and sexually transmitted infections. International journal of STD & AIDS. 2007;18(11):764-9.

24. Kotlewski JA, Kimaru L, Sharkey T, Oppert MA, Kilembe W, Inambao M, et al. HIV and Sexually Transmitted Infection (STI) Testing among Female Sex Workers (FSWs) in Urban Zambia. AIDS research and human retroviruses. 2014;30(S1):A134-A.

25. Choongo M. Syphilis infection among female sex workers and single women in Lusaka: perceptions, practices and associated factors: The University of Zambia; 2019.
